# The Dilemma of *TP53* Codon 72 Polymorphism (rs1042522) and Breast Cancer Risk: A Case-Control Study and Meta-Analysis in The Iranian Population

**DOI:** 10.22074/cellj.2020.6458

**Published:** 2019-10-14

**Authors:** Fahimeh Afzaljavan, Negin Chaeichi Tehrani, Mahdi Rivandi, Saeed Zarif Ghasemian, Elham Vahednia, Reza Khayami, Mohammad Abavisani, Alireza Pasdar

**Affiliations:** 1. Department of Modern Sciences and Technologies, Faculty of Medicine, Mashhad University of Medical Science, Mashhad, Iran; 2. Student Research Committee, Faculty of Medicine, Mashhad University of Medical Sciences, Mashhad, Iran; 3. Department of Radiology Technology, Faculty of Paramedical Sciences, Mashhad University of Medical Sciences, Mashhad, Iran; 4. Department of Medical Genetics, Faculty of Medicine, Mashhad University of Medical Science, Mashhad, Iran; 5. Division of Applied Medicine, Medical School, University of Aberdeen, Foresterhill, Aberdeen, AB25 2ZD, UK

**Keywords:** Breast Cancer, Genetic Variation, Polymorphism, *TP53*

## Abstract

**Objective:**

Mutations of TP53 as a tumor suppressor gene are frequently observed in different types of cancer. A codon 72 polymorphism located on exon 4 with two alleles encoding either Proline (CCC) or Arginine (CGC) has been indicated as a common variation in association with cancers. Controversial results have been reported regarding the association of allelic polymorphism of codon 72 of *TP53* gene and breast cancer risk in Iranian patients. Therefore, a case-control study was designed. A meta-analysis was also carried out to provide evidence of association between this variation and breast cancer in Iran, based on all available published data.

**Materials and Methods:**

In this case-control study, blood sample of 622 participants, including 308 breast cancer cases and 314 controls were collected. Genotyping for rs1042522 was conducted by Allele Specific polymerase chain reaction (AS-PCR). In order to set a meta-analysis study, PubMed, Scopus and ISI Web of Knowledge and Persian databases were searched to explore relevant studies, published up to September 2018, containing information on *TP53* polymorphism and the risk of breast cancer in Iran. Statistical analysis was performed using SPSS 16.0 and MetaGenyo.

**Results:**

All retrieved available data as well as the results of our current study were consisted of 1965 breast cancer cases and 1999 healthy controls. No significant difference was observed in allele frequencies between groups (P=0.90) in our study. The cumulative results did not also show any association between rs1042522 and breast cancer risk on the dominant (P=0.61) and recessive (P=0.89) models.

**Conclusion:**

These findings cannot support contribution of rs1042522 polymorphism to breast cancer risk in an Iranian population. Future larger studies may help confirm this finding with a greater power. .

## Introduction

Breast malignancy is among the major types of cancer and the universal cause of cancer death in women. The incidence of breast cancer in western populations is significantly higher than other populations. However, 50% of new cases and approximately 60% of deaths caused by breast cancer occur in developing countries (1). Breast cancer is one of the most commonest cancers affecting Iranian women, though the epidemiology of breast cancer in Iran has not yet been fully investigated (2).

Based on epidemiological studies, a number of factors have been identified associating with increased risk of breast malignancies. These factors are not necessarily causes of breast cancer and they can be subcategorized in both genetic and environmental factors, increasing liability of the breast cancer. Association studies, whereby the relations of certain markers with the disease are investigated prominently in case-control designs, are of crucial importance to dissect the genetic basis of common multifactorial disorders, such as breast cancer. Based on this, several candidate genes have been analyzed so far in case-control studies.

One of the highest involved genetic factors in the risk of breast cancer is the tumor suppressor gene, TP53. The corresponding protein, P53, has a role in cell cycle regulation including cell growth and division, apoptosis, DNA repair and the maintenance of genome stability and its mutations have been commonly observed in different types of cancer (3, 4). Dysfunction of the P53 signaling pathway is an important hallmark of different malignancies (5). Numerous single nucleotide polymorphisms (SNPs), somatic and germ line mutations are located at the TP53 locus. Polymorphisms in this gene may affect the susceptibility to cancer development in the way of varying the normal functions of P53. Since many of these variations have been found in intronic loci, they cannot affect the biology of cancer. However, they may be used as markers in dissecting genetic basis of multifactorial diseases. Previously, limited numbers of TP53 polymorphisms have been studied in relation with biochemical and biological functions and in association with cancer risk. The TP53 codon 72 polymorphism (C/G) has been located at the exon 4 of this gene and encodes Proline (CCC) or Arginine (CGC). Association of this polymorphism with susceptibility to several forms of cancer has been identified (6). According to research on different populations, genotype frequencies have been found to largely differ with ethnicity changes (7). The difference in the primary structure of P53 protein results in different biochemical functions. The potential role of Arg variant in the induction of apoptosis pathway has been confirmed in a previous research (8). Furthermore, "Pro" variant can block cell cycle pathway progression toward the repair of DNA damage (9).

Association of the *TP53* codon 72 polymorphism and susceptibility to breast cancer has been considered in several regions of Iran. These case-control studies did not indicate consistent results, likely due to the small sample sizes with limited power. A meta-analysis along with combining the different results and small data, can achieve a reasonable level of significance and an increase in power of results. In the present study, we examined association of rs1042522 and breast cancer susceptibility in the North-East of Iran and a meta-analysis was performed to quantitatively assess effect of the *TP53* codon 72 polymorphism on risk of breast cancer in Iran.

## Materials and Methods

### Population study

In this case-control study, 308 breast cancer cases and 314 healthy controls with no sign of breast cancer or history of malignant breast disease participated in this association study. The important demographics and histopathological data were obtained from a questionnaire. The Ethics Committee of the Mashhad University of Medical Science approved this study (ethical approval number: IR.MUMS. fm.REC.1394.472) and all of the participants signed the written informed consent.

### DNA extraction and genotyping analysis

The salting out method was used to extract DNA from peripheral blood samples. Genotyping was performed using Allele specific polymerase chain reaction (AS-PCR) methods (10). We used primers from the previous study (11) and the sequence of four primers (synthesis by metabion international AG, Germany) was shown as follows:

Arginine-based (G) allele:

F: 5´-TCCCCCTTGCCGTCCCAA-3´

R: 5´-CTGGTGCAGGGGCCACGC-3´

Proline-based (C) allele:

F: 5´-GCCAGAGGCTGCTCCCCC-3´

R: 5´-CGTGCAAGTCACAGACTT-3´

PCR was done in a final volume of 10 μl reaction for each allele containing: Taq DNA Polymerase 2x Master Mix RED (Ampliqon, Denmark), 1 μl genomic DNA (200-300 ng), 1 μl of each primer (10 μM, Metabion International AG, Germany) and adequate DNase free water (Sinaclon, Iran). Amplification temperature stages were performed for 5 minutes at 95˚C and then 35 cycles including 30 seconds at 94˚C, 30 seconds at 63˚C, 30 seconds at 72˚C, followed by 7 minutes at 72˚C in a Veriti 96 well PCR Thermal Cycler (Thermo Fisher Scientific).

### Identiﬁcation of studies for meta-analysis

The original publications, reporting association between *TP53* codon 72 polymorphism and breast cancer risk before September 2018, were gathered by searching Scopus, PubMed and ISI Web of Knowledge databases. The Persian articles and conference abstracts were also explored by searching SID, Iranmedex, Magiran, Medlib and Google. The references of retrieved articles were also investigated to discover other related studies. The terms of "TP53" and "genetic variant", "genetic variation" or "polymorphism" and "breast cancer", "breast carcinoma", "breast tumour" or "breast tumor" and "Iran" or "Iranian" were used to search for the articles of interest. Case-control studies were selected for the analysis.

### Data extraction

A meta-analysis was designed according to PRISMA guidelines (12). Features of the selected studies were independently extracted by two authors. For each eligible study, first author, date of publication, number of cases and controls, study population (region), frequency of all genotypes for two groups, allele incidence and Hardy- Weinberg equilibrium (HWE) in control groups were extracted or calculated and ultimately the results were reviewed by the third investigator.

### Statistical analysis

Chi-square test and logistic regression models were performed to find association between histopathological or demographic criteria and genotypes of rs1042522.

In the meta-analysis study, frequency of two alleles was calculated for the case and control groups in each experiment. HWE test using the X^2^ statistic was evaluated by analysis of the genotype frequencies in the controls. Association was measured initially using both random-effect and fixed-effect models. However, since the random-effect method proposes heterogeneity, this method was considered as the main approach. The strength of association between TP53 codon 72 polymorphism and risk of breast cancer was measured by odds ratios (ORs) with 95% confidence intervals (CIs).

The risk for the genotypes GC and CC was compared to the GG homozygote, as the wild-type genotype. Furthermore, according to the dominant and recessive models, the risk of Arg-carriers (GC+GG) versus CC genotype and Pro-carriers (CC+GC) versus GG genotype were evaluated, respectively.

Heterogeneity was evaluated by the Q-test and I^2^ index. Probability of publication bias was checked by the funnel plot and Egger’s test for all genetic models. Random effects model was used to analyze the data.

Meta-analysis was carried out using MetaGenyo [Pfizer- University of Granada-Junta de Andalucía Centre for Genomics and Oncological Research (GENYO), Spain] (13). All other statistical analyses were carried out using SPSS version 16.0 (SPSS Inc., USA). A P value of less than 0.05 was considered statistically significant.

## Results

### Characteristics of the population study

A total of 308 breast cancer women, out of 622 samples, and 314 healthy controls were enrolled in this study. The average age of case and control groups were 47.80 ± 10.90 and 44.15 ± 12.07 years, respectively, with a significant difference between the groups (P<0.001). Furthermore, menopausal status was considered as peri- and pre-menopausal, compared to postmenopausal individuals. The control group was younger than cases, and therefore most of the patients typically belonged to the post-menopausal group. The difference between groups was significant (P<0.001).

Body mass index (BMI), as a continuous and categorical variable, was compared between these groups. Mean BMI was higher in the patient cases than in controls and a significant difference was observed (P<0.001). Moreover, by categorizing patients, it was distinguished that a large number of cases were included in BMI ≥25 category.

The history of lactation (P<0.01), history of other cancers (P<0.001) and family history of cancer (P<0.001) were also significantly different between patients and healthy controls. Table 1 shows the most important demographic characteristics of the case and control groups.

### Association between rs1042522 and the risk of breast cancer in the Northeast of Iran

Genotypic distribution of the rs1042522 in our study conforms HWE (P>0.05). The C allele (Pro) frequency, as the minor allele was 24% in the cases, compared to 23.7% in the controls. According to this data, no association was found between case and control groups for allele frequency (P=0.90). The most frequent genotype for rs1042522 was GG (Arg/Arg) in both case (60.7%) and control (60.2%) groups. Genotype frequency did not show any significant difference between these two groups (P>0.05). Moreover, dominant and recessive models did not indicate any association with the risk of breast cancer. Results have been shown in Table 2.

Adjustment for confounding factors including age, BMI, history of cancer, family history of cancer and history of lactation did not change the results.

### Meta-analysis

**Fig 1:**
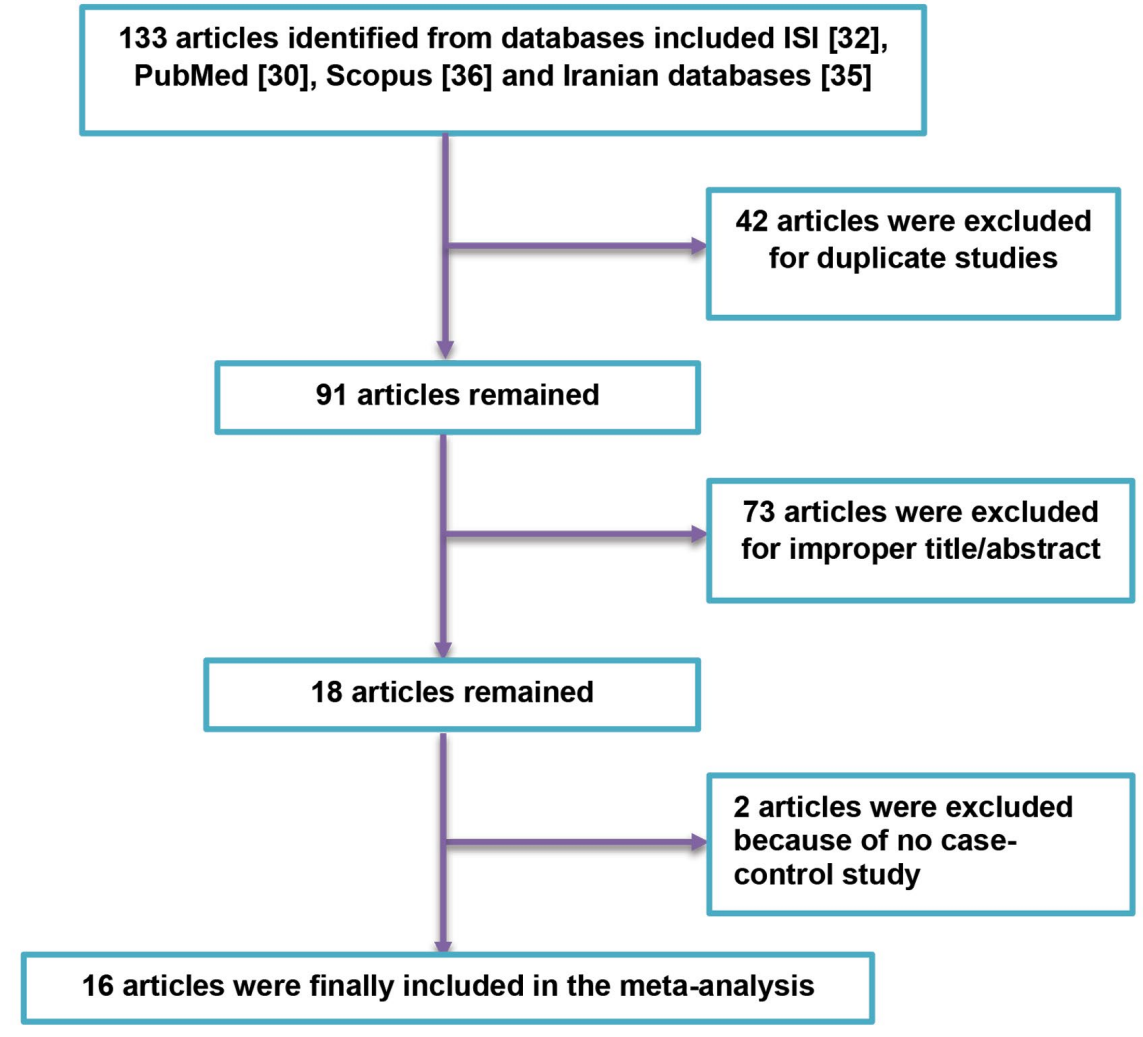
Flowchart of the search strategy and selection of studies. After searching in main databases, with removing 117 studies due to duplication, improper subjects and no case-control design, 16 articles remanied to be included in the mata-analysis.

133 articles were identified from different databases by two authors, individually. After matching the data, discrepancies were reanalyzed by the third author. 42 articles were similar between databases. Therefore, 91 articles were assessed for eligibility. 73 studies were not related to the subject. Furthermore, two records were not designed as the case-control study. After excluding duplicated, unrelated and improperly designed papers, 16 case-control studies, concerning the association between TP53 codon 72 polymorphism and breast cancer, were included in the meta-analysis. The selection process has been demonstrated in Figure 1.

**Table 1:** Results of the association analysis of demographic characteristics between breast cancer cases and healthy group


Characteristics	Cases	Controls	P value
	n (%)	n (%)	

Age (Y)			
≤40	74 (25.8)	138 (44.4)	
>40	213 (74.2)	173 (55.6)	<0.001
Mean	47.80 ± 10.90	44.15 ± 12.07	<0.001
Menopausal status			
Peri and premenopausal	86 (45.3)	217 (78.9)	
Postmenopausal	104 (54.7)	58 (21.1)	<0.001
Body mass index (kg/m^2^)			
<25	80 (31.2)	154 (51.2)	
≥25	176 (68.8)	147 (48.8)	<0.001
Mean	27.59 ± 5.06	25.20 ± 4.12	<0.001
Abortion			
Yes	172 (67.5)	151 (65.1)	
No	83 (32.5)	81 (34.9)	0.63
History of lactation			
Yes	246 (91.4)	223 (97.4)	
No	23 (8.6)	6 (2.6)	<0.01
Family history of cancer			
Yes	172 (60.8)	229 (73.9)	
No	111 (39.2)	81 (26.1)	<0.001
History of other cancer			
Yes	20 (7.2)	3 (1.0)	
No	259 (92.8)	301 (99.0)	<0.001


Data are presented as mean ± SD or n (%).

**Table 2:** Distribution of the genotypes and allele frequency of rs1042522 polymorphism in breast cancer cases and controls


Genetic analysis model	Number of case (%)	Number of control (%)	P value	OR (95% CI)

Genotypes				
GG	187 (60.7)	189 (60.2)	Reference	
GC	94 (30.5)	101 (32.2)	0.73	0.94 (0.67-1.33)
CC	27 (8.8)	24 (7.6)	0.67	1.38 (0.63-2.04)
Dominant				
GG+GC	281 (91.2)	290 (92.4)	Reference	
CC	27 (8.8)	24 (7.6)	0.61	0.86 (0.48-1.53)
Recessive				
GG	187 (60.7)	189 (60.2)	Reference	
GC+CC	121 (39.3)	125 (39.8)	0.89	0.98 (0.71-1.35)
Allele				
G	468 (76.0)	479 (76.3)	Reference	
C	148 (24.0)	149 (23.7)	0.90	1.02 (0.78-1.32)


OR; Odd ratio and CI; Confidence interval.

### Data extraction

Overall the data included 1965 breast cancer subjects and 1999 healthy people. Diversity in the sample size, ranged from 42 to 314 individuals, was found between studies. Moreover, genotyping methods were different between projects. Nine studies used the AS-PCR method and seven studies were performed by the PCR-restriction fragment length polymorphism (PCR-RFLP) method. Although the main source of DNA template was blood in many studies, one study had been performed on normal and cancerous tissues and five projects had used tissue samples for patient’s genotyping and blood samples for healthy groups.

Frequency of the 72 Pro allele varied in the control participants, from 30 to 60%, depending on the geographical region. Apart from the four studies, HWE was confirmed for frequency of *TP53* codon 72 genotypes in the control group. The results have been shown in Table 3.

Pooled allele frequencies did not significantly differ between Iranian patients and healthy controls. Results confirmed a lack of association between the TP53 codon 72 and susceptibility to breast cancer in all genetics models. This finding did not vary after removing studies with no HWE data.

Publication bias was evaluated by the analysis of Begg’s funnel plots and Egger’s test for all genetic models. All plots indicated some evidence of publication bias, however, this finding was not significant (P>0.05, plots have not been shown). According to the P value of heterogeneity, we found high incidence of heterogeneity between the studies in different genetic models (P<0.01). Results have been shown in Table 4.

**Table 3:** Extracted data from the selected studies


Study	Year	Region	Type of samples		Method	Sample size		Genotype frequency						p HWE	Allele frequency (%)			
								Cases			Controls				Cases		Controls	
			Cases	Controls		Cases	Controls	A/A	A/P	P/P	A/A	A/P	P/P		G	C	G	C

Faghani et al. (14)	2007	Isfahan	Tissue	Blood	AS-PCR	51	51	44	6	1	22	27	2	0.19	92.1	7.8	69.6	30.4
Khadang et al. (15)	2007	Shiraz	Blood	Blood	AS-PCR	221	205	83	109	29	75	90	40	0.39	62.2	37.8	58.5	41.5
Faghani et al. (16)	2008	Isfahan	Tissue	Blood	AS-PCR	96	96	68	21	7	35	44	17	0.88	81.7	18.2	59.4	40.6
Kazemi et al. (17)	2009	North of Iran	Tissue	Blood	AS-PCR	42	60	6	30	6	12	45	0	0	50	50	39.5	60.5
Doosti et al. (18)	2011	Isfahan	Blood	Blood	PCR-RFLP	135	140	52	70	13	36	82	22	0.09	64.4	35.6	55	45
Hossein Pour Feizi et al. (19)	2012	Tabriz	Blood	Blood	AS-PCR	126	99	56	44	26	30	50	19	0.97	61.9	38.1	55.6	44.4
Golmohammadi and Namazi (20)	2013	Sabzevar	Blood	Blood	AS-PCR	80	80	29	49	2	15	51	14	0.04	66.8	33.2	50.6	49.4
Rouhi Boroujeni et al. (21)	2013	Isfahan	Blood	Blood	PCR-RFLP	135	150	27	102	6	36	93	21	0.01	57.8	42.2	55	45
Behfarjam et al. (22)	2013	Mahabad	Blood	Blood	PCR-RFLP	25	30	9	14	2	9	17	4	0.66	64	36	58.3	42.7
Sheikhpour and Taghipour Zahir (23)	2014	Yazd	Blood	Blood	AS-PCR	104	104	51	31	22	22	54	28	0.91	63.9	36.1	47.1	52.9
Saadatian et al. (24)	2014	Tabriz	Blood	Blood	PCR-RFLP	100	100	22	48	30	13	63	24	0.02	46	54	44.5	55.5
Gohari-Lasaki et al. (25)	2015	Tabriz	Blood	Blood	PCR-RFLP	100	100	31	48	21	31	57	12	0.18	55	45	59.5	40.5
Ahangar Oskouee et al. (26)	2015	Tabriz	Tissue	Tissue	PCR-RFLP	65	65	21	40	4	48	13	4	0.11	63.1	36.9	83.8	16.2
Rajabi Firoozabadi et al. (27)	2016	Yazd	Blood	Blood	AS-PCR	90	83	10	45	35	21	37	25	0.62	36.1	63.9	47.5	52.5
Moradinasab et al. (28)	2017	Bushehr	Blood	Blood	PCR-RFLP	144	162	46	68	30	50	90	22	0.18	55.6	44.4	58.6	41.4
Pouladi et al. (29)	2018	Tabriz	Tissue	Blood	AS-PCR	143	160	63	54	26	54	74	32	0.77	62.9	37.1	56.8	43.2
Our study	2018	Northeast of Iran	Blood	Blood	AS-PCR	308	314	187	94	27	189	101	24	0.14	76	24	76.2	23.8
Total (17 studies)						1965	1999	805	873	287	698	988	310	0.43	63.4	36.6	59.7	40.3


AS-PCR; Allele specific polymerase chain reaction, RFLP; Restriction fragment length polymorphism, and HWE; Hardy-Weinberg.

**Table 4:** Analysis of the association between TP53 codon 72 polymorphism and breast cancer risk in different genetic models, the test of heterogeneity and publication bias


Model	Test of association		Test of heterogeneity			Publication bias
	OR (95% CI)	P value	Model	P value	I^2^	P value (Egger’s test)

Allele contrast (G vs. C)	1.18 (0.96-1.47)	0.13	Random	<0.001	0.80	0.63
Recessive model (GG vs. GC+CC)	1.34 (0.95-1.89)	0.10	Random	<0.001	0.83	0.73
Dominant model (GG+GC vs. CC)	1.15 (0.84-1.56)	0.38	Random	<0.01	0.58	0.51
Over dominant (GC vs. GG + CC)	0.79 (0.58-1.06)	0.12	Random	<0.001	0.79	0.50
Pairwise 1 (GG vs. CC)	1.38 (0.93-2.04)	0.11	Random	<0.001	0.67	0.83
Pairwise 2 (GG vs. GC)	1.36 (0.95-1.95)	0.10	Random	<0.001	0.82	0.66
Pairwise 3 (GC vs. CC)	1.00 (0.72-1.36)	0.97	Random	<0.01	0.55	0.63


OR; Odd ratio and CI; Confidence interval.

## Discussion

The mortality rate in comparison with the rate of breast cancer is higher in developing rather than developed countries, due to the early detection of diseases using genetic biomarkers in the latter countries. SNPs are a good example of these biomarkers. The TP53 codon 72 polymorphism (rs1042522) has been reported to have an association with the susceptibility to cancer in the different populations. Due to the lack of association studies with sufficient sample sizes and strong conclusions to assess correlation between rs1042522 and risk of breast cancer development, we conducted the case-control study on 622 breast cancer patients and controls in the northeast of Iran.

A previous meta-analysis study without any ethnicity restriction showed association of TP53 codon 72 polymorphism with breast cancer risk in the recessive model. This study only confirmed a significant difference in the allelic model of an Asian population (14). However, involvement of other risk factors in this association was suggested. Genetic background and ethnicity are amongst those influential factors that may change association status. Since several case-control studies have reported different results about the association between rs1042522 and breast cancer risk in Iran, the need for a comprehensive analysis was warranted. In order to elucidate this inconsistent conclusion, a meta-analysis was performed to examine the association between TP53 codon 72 polymorphism and breast cancer risk in Iran, by reviewing all published studies with conflicting results. The final analysis included a total of 1965 breast cancer patients and 1999 healthy individuals, as the control group, to evaluate the existence or absence of any association.

Our pooled data from 17 case-control studies, meeting the inclusion criteria, confirmed lack of association between risk of breast cancer and TP53 codon 72 polymorphism in Iran. The heterogeneity amongst the studies has been pointed out in the analysis. Ethnicity, minor allele frequency, sample size, genotyping methods, source of DNA and disease phenotypes may be regarded as the source of such heterogeneity. However, we acknowledge that we focused on studies considering germline variants as risk factors, there are some studies which may have investigated the somatic variants to provide a catalogue of tumoral genetic variations (15, 16).

Our results indicated that pooled control samples followed HWE. Four studies failed to show HWE, however, when they were excluded from the analysis, the overall results were not significantly changed (data has been shown in the Table S1) (See Supplementary Online Information at www.celljournal.org).

Pooled allele frequencies were 0.60 and 0.63 for G in the control and case groups, respectively. According to NCBI and OMIM data, C allele is the ancestral allele coding Proline. Allele frequency of C is higher than G in the general population, however, population based studies indicate high heterogeneity related to the C and G allele frequencies. According to some online databases, the frequencies vary between 0.9 for C and 0.1 for G in Oceania and inversely 0.2 and 0.8 for C and G respectively in Europe, suggesting a powerful ethnic influence (17). In our study, minor allele was C allele in the pooled data with a frequency of 0.37 and 0.40 in the respectively controls and cases.

The results did not show a significant difference between allele frequencies in patients and healthy subjects (P in multiplicative model ≤0.12). This finding was also seen in recessive and dominant models. Statistical evidence from other meta-analysis studies have indicated this polymorphism may have a potential effect on breast (14) and ovarian cancer risks (18) in the pooled data as well as a subgroup of Asian and Caucasian populations. On the other hand, in another previously conducted metaanalysis (with no ethnicity preference) no association between TP53 codon 72 polymorphism with breast cancer (19) and cervical cancer (20) risks was reported.

The pooled data indicated that allele frequency difference between cases and controls was about 3%. Therefore, it is proposed to evaluate the association between TP53 codon 72 polymorphism and risk of breast cancer with a power of 80%; in other words 4129 individuals is proposed for each group in future studies. In our study the overall power was calculated as 50% which may not support the evidence of this association. Designing proper studies with adequate sample sizes will provide more valuable evidence for this association.

## Conclusion

Our results showed that TP53 codon 72 polymorphism may not influence the overall risk of breast cancer in an Iranian population. By that means, there is no association between TP53 codon 72 polymorphism and breast cancer risk in the Iranian population of this meta-analysis. Several included studies had limited sample size and they were underpowered. To assess this association more precisely, well-designed case-control studies with adequate sample sizes are still necessary.

Molecular classification, as well as evaluating the effect of other polymorphisms and environmental factors, such as alcohol consumption and tobacco smoke, should also be considered. Characteristics of different individuals, including premenopausal or postmenopausal status, metabolic index, family history, epistasis and clinical course are also important.

## Supplementary PDF



## References

[1] Forouzanfar MH, Foreman KJ, Delossantos AM, Lozano R, Lopez AD, Murray CJ (2011). Breast and cervical cancer in 187 countries between 1980 and 2010: a systematic analysis. Lancet.

[2] Montazeri A, Vahdaninia M, Harirchi I, Harirchi AM, Sajadian A, Khaleghi F (2008). Breast cancer in Iran: need for greater women awareness of warning signs and effective screening methods. Asia Pac Fam Med.

[3] Green DR, Kroemer G (2009). Cytoplasmic functions of the tumour suppressor p53. Nature.

[4] Muller PA, Vousden KH (2013). p53 mutations in cancer. Nat Cell Biol.

[5] Vogelstein B, Lane D, Levine AJ (2000). Surfing the p53 network. Nature.

[6] Whibley C, Pharoah PD, Hollstein M (2009). p53 polymorphisms: cancer implications. Nat Rev Cancer.

[7] Sjalander A, Birgander R, Saha N, Beckman L, Beckman G (1996). p53 polymorphisms and haplotypes show distinct differences between major ethnic groups. Hum Hered.

[8] Dumont P, Leu JI, Della Pietra AC 3rd, George DL, Murphy M (2003). The codon 72 polymorphic variants of p53 have markedly different apoptotic potential. Nat Genet.

[9] Siddique M, Sabapathy K (2006). Trp53-dependent DNA-repair is affected by the codon 72 polymorphism. Oncogene.

[10] Suguna S, Nandal DH, Kamble S, Bharatha A, Kunkulol R (2014). Genimic DNA isolation from human whole blood samples by non enzymatic salting out method. International Journal of Pharmacy and Pharmaceutical Sciences.

[11] Storey A, Thomas M, Kalita A, Harwood C, Gardiol D, Mantovani F (1998). Role of a p53 polymorphism in the development of human papillomavirus-associated cancer. Nature.

[12] PRISMA (2015). http://www.prisma-statement.org/.

[13] Martorell-Marugan J, Toro-Dominguez D, Alarcon-Riquelme ME, Carmona-Saez P (2017). MetaGenyo: A web tool for meta-analysis of genetic association studies. BMC Bioinformatics.

[14] Faghani M, Nikbahkt M, Salehi M, Rabbani M, Talebi A, Soleimani B (2007). Study of p53 polymorphism at codon 72 in patients of breast cancer in Isfahan. Journal of Isfahan Medical School.

[15] Khadang B, Fattahi MJ, Talei A, Dehaghani AS, Ghaderi A (2007). Polymorphism of TP53 codon 72 showed no association with breast cancer in Iranian women. Cancer Genet Cytogenet.

[16] Faghani M, Nasiri E, Bahadori MH, Mohammad Ghasemi F (2008). Genetic predisposing of p53 codon 72 on developing of breast cancer in postmenopausal women in Isfahan. Journal of Guilan University of Medical Sciences.

[17] Kazemi M, Salehi Z, Chakosari RJ (2009). TP53 codon 72 polymorphism and breast cancer in northern Iran. Oncol Res.

[18] Doosti A, Ghasemi Dehkordi P, Davoudi N (2011). A p53 codon 72 polymorphism associated with breast cancer in Iranian patients. Afr J Pharm Pharmacol.

[19] Hossein Pour Feizi MA, Ravanbakhsh R, Pourahmad R, Pouladi N, Azarfam P, Montazeri V (2012). Association of p53 Arg/Pro polymorphism at codon 72 with Risk of breast cancer in East Azerbaijani women. J Babol Univ Med Sci.

[20] Golmohammadi R, Namazi MJ (2013). The role of p53 codon 72 genotype in ductal breast carcinoma. Feyz.

[21] Rouhi Boroujeni H, Karimi M, Moshkelani S, Parsaei P (2013). Association of the p53 codon 72 polymorphism with breast cancer in central part of Iran. Afr J Pharm Pharmacol.

[22] Behfarjam F, Rostamzadeh J, Faegh J (2013). Study of TP53 codon 72 & 282 polymorphisms and ABCC1 promoter polymorphism (G- 1666A) in archived breast cancer samples mahabadian women by PCR-RFLP and PCR-SSCP.8th International Breast Cancer Congress; 2013; Tehran, Iran.Tehran, Iran; Shahid Beheshti University of Medical Sciences.

[23] Sheikhpour R, Taghipour Zahir Sh (2014). Analysis of P53 codon 72 polymorphism and protoein level in breast cancer patients in Yazd. Iranian Journal of Breast Diseases.

[24] Saadatian Z, Gharesouran J, Ghojazadeh M, Ghohari-Lasaki S, Tarkesh-Esfahani N, Mohaddes Ardebili SM (2014). Association of rs1219648 in FGFR2 and rs1042522 in TP53 with premenopausal breast cancer in an Iranian Azeri population. Asian Pac J Cancer Prev.

[25] Gohari-Lasaki S, Gharesouran J, Ghojazadeh M, Montazeri V, Mohaddes Ardebili SM (2015). Lack of influence of TP53 Arg72Pro and 16bp duplication polymorphisms on risk of breast cancer in Iran. Asian Pac J Cancer Prev.

[26] Ahangar Oskouee M, Shahmahmoudi S, Nategh R, Esmaeili HA, Safaeyan F, Zareinghalame Moghaddam M (2015). Three common TP53 polymorphisms and the risk of breast cancer among groups of iranian women.. Arch Breast Cancer.

[27] Rajabi Firoozabadi S, Shiryazdi SM, Keshavarz F, Nazari T, Ghasemi N (2016). Frequency of p53 gene codon 72 polymorphisms in women with breast cancer in Iran. IJML.

[28] Moradinasab M, Ostovar A, Nabipour I, Eghbali SS, Vahdat K, Ghaderi A (2017). TP53 codon 72 genetic polymorphism, rs1042522, Modifies the association between tobacco smoking and breast cancer risk. Iran Red Crescent Med J.

[29] Pouladi N, Dehghan R, Hosseinpour Feizi M, Dastmalchi N (2018). Association of P53 (+16ins-Arg) haplotype with the increased susceptibility to breast cancer in Iranian-Azeri women. Journal of Kerman University of Medical Sciences.

[30] Gonçalves ML, Borja SM, Cordeiro JA, Saddi VA, Ayres FM, Vilanova-Costa CA (2014). Association of the TP53 codon 72 polymorphism and breast cancer risk: a meta-analysis. Springerplus.

[31] ALFRED (2019, updated 2002). http://alfred.med.yale.edu/alfred/SiteTable1A_working.asp?siteuid=SI018437X.

[32] Zhang Z, Fu G, Wang M, Tong N, Wang S, Zhang Z (2008). P53 codon 72 polymorphism and ovarian cancer risk: a meta-analysis. Journal of Nanjing Medical University.

[33] Hou J, Jiang Y, Tang W, Jia S (2013). p53 codon 72 polymorphism and breast cancer risk: a meta-analysis. Exp Ther Med.

[34] Klug SJ, Ressing M, Koenig J, Abba MC, Agorastos T, Brenna SM (2009). TP53 codon 72 polymorphism and cervical cancer: a pooled analysis of individual data from 49 studies. Lancet Oncol.

