# Supplementary material for: The Dilemma of TP53 Codon 72 Polymorphism (rs1042522) and Breast Cancer Risk: A Case-Control Study and Meta-Analysis in The Iranian Population
Source: Cell J. 2019 Oct 14;22(2):185–92. doi: 10.22074/cellj.2020.6458 (PMC6874791; doi:10.22074/cellj.2020.6458)
Supplement: Supplementary file 1 [file Cell-J-22-185-s01.pdf]

CELL JOURNAL (Yakhteh)

Supplementary Information for

The Dilemma of *TP53* Codon 72 Polymorphism (rs1042522) and Breast Cancer Risk: A Case-Control Study and Meta-Analysis in The Iranian Population

Fahimeh Afzaljavan, Ph.D.[1, 2#], Negin Chaeichi Tehrani, B.Sc.[1#], Mahdi Rivandi, D.V.M.[1, 2#], Saeed Zarif Ghasemian, B.Sc.[2, 3], Elham Vahednia, M.Sc.[1], Reza Khayami, B.Sc.[4], Mohammad Abavisani, B.Sc.[2], Alireza Pasdar, M.D., Ph.D.[1, 5*]

1. Department of Modern Sciences and Technologies, Faculty of Medicine, Mashhad University of Medical Science, Mashhad, Iran
2. Student Research Committee, Faculty of Medicine, Mashhad University of Medical Sciences, Mashhad, Iran
3. Department of Radiology Technology, Faculty of Paramedical Sciences, Mashhad University of Medical Sciences, Mashhad, Iran
4. Department of Medical Genetics, Faculty of Medicine, Mashhad University of Medical Science, Mashhad, Iran
5. Division of Applied Medicine, Medical School, University of Aberdeen, Foresterhill, Aberdeen, AB25 2ZD, UK

#The first three authors equally contributed to this work.

**Corresponding Address: P.O.Box: 917794-8564, Department of Modern Sciences and Technologies, Faculty of Medicine, Mashhad University of Medical Science, Mashhad, Iran*
Email: pasdara@mums.ac.ir

Table S1: Subgroup analysis using different models of association between TP53 codon 72 polymorphism and breast cancer risk based on Hardy-Weinberg equilibrium (HWE) results

Model	HWE	Number of studies	Test of association		Test of heterogeneity			Publication bias	
			OR	95% CI	P value	Model	P value	I^2	P value (Egger's test)
Allele contrast (A vs. a)	Overall	17	1.1845	[0.9526; 1.4729]	0.127617264	Random	0.0001	0.8027	0.6291
	Yes	13	1.2051	[0.9231; 1.5733]	0.170215831	Random	0.0001	0.8326	0.5656
	No	4	1.1356	[0.7784; 1.6567]	0.50927386	Random	0.0241	0.6818	0.7643
Recessive model (AA vs. Aa+aa)	Overall	17	1.3388	[0.9465; 1.8938]	0.099073097	Random	0.0001	0.8284	0.7329
	Yes	13	1.3611	[0.9013; 2.0554]	0.142677594	Random	0.0001	0.858	0.7131
	No	4	1.2699	[0.6706; 2.4048]	0.463350766	Random	0.0342	0.6536	0.9418
Dominant model (AA+Aa vs. aa)	Overall	17	1.1474	[0.8451; 1.5577]	0.378251142	Random	0.0016	0.5766	0.5065
	Yes	13	1.0586	[0.8146; 1.3758]	0.669969798	Random	0.0888	0.368	0.5248
	No	4	1.4286	[0.3306; 6.1732]	0.63287075	Random	0.0004	0.8335	0.92
Overdominant (Aa vs. AA + aa)	Overall	17	0.7866	[0.5831; 1.0612]	0.116152629	Random	0.0001	0.7949	0.5063
	Yes	13	0.7527	[0.5302; 1.0686]	0.112085206	Random	0.0001	0.8159	0.5467
	No	4	0.9119	[0.4912; 1.6931]	0.770203977	Random	0.0101	0.7348	0.551
pairw1 (AA vs. aa)	Overall	17	1.3757	[0.9263; 2.0433]	0.113984034	Random	0.0001	0.6752	0.8269
	Yes	13	1.2587	[0.8440; 1.8771]	0.259304476	Random	0.0006	0.6486	0.8334
	No	4	1.798	[0.4320; 7.4832]	0.420007618	Random	0.0049	0.767	0.7511
pairw2 (AA vs. Aa)	Overall	17	1.3577	[0.9472; 1.9462]	0.096015543	Random	0.0001	0.8216	0.6588
	Yes	13	1.394	[0.9070; 2.1424]	0.129850986	Random	0.0001	0.8512	0.6737
	No	4	1.2387	[0.6444; 2.3811]	0.520946726	Random	0.0337	0.6548	0.7147
pairw3 (Aa vs. aa)	Overall	17	0.9933	[0.7242; 1.3624]	0.966717319	Random	0.003	0.5547	0.6266
	Yes	13	0.9062	[0.7328; 1.1205]	0.362969116	Fixed	0.2118	0.2293	0.7386
	No	4	1.3031	[0.2809; 6.0456]	0.735295712	Random	0.0002	0.8438	0.9152
